# A novel mouse model for N-terminal truncated Aβ2-x generation through meprin β overexpression in astrocytes

**DOI:** 10.1007/s00018-024-05139-w

**Published:** 2024-03-13

**Authors:** Fred Armbrust, Kira Bickenbach, Hermann Altmeppen, Angelica Foggetti, Anne Winkelmann, Peer Wulff, Markus Glatzel, Claus U. Pietrzik, Christoph Becker-Pauly

**Affiliations:** 1 Biochemical Institute, Unit for Degradomics of the Protease Web, University of Kiel, Otto-Hahn-Platz 9, 24118 Kiel, Germany; 2 Institute of Neuropathology, University Medical Center Hamburg-Eppendorf, Hamburg, Germany; 3 Institute of Physiology, University of Kiel, Kiel, Germany; 4 Zhejiang University-University of Edinburgh Institute, Zhejiang University School of Medicine, Haining, 314400 China; 5 College of Medicine & Veterinary Medicine, The University of Edinburgh, Edinburgh, United Kingdom; 6 Institute for Pathobiochemistry, University Medical Center of the Johannes Gutenberg University Mainz, Mainz, Germany

**Keywords:** Alternative β-secretase, Alzheimer’s disease, Amyloid-β, APP, Astrocytes, Meprin β

## Abstract

Neurotoxic amyloid-β (Aβ) peptides cause neurodegeneration in Alzheimer’s disease (AD) patients’ brains. They are released upon proteolytic processing of the amyloid precursor protein (APP) extracellularly at the β-secretase site and intramembranously at the γ-secretase site. Several AD mouse models were developed to conduct respective research in vivo. Most of these classical models overexpress human APP with mutations driving AD-associated pathogenic APP processing. However, the resulting pattern of Aβ species in the mouse brains differs from those observed in AD patients’ brains. Particularly mutations proximal to the β-secretase cleavage site (e.g., the so-called Swedish APP (APPswe) fostering Aβ1-x formation) lead to artificial Aβ production, as N-terminally truncated Aβ peptides are hardly present in these mouse brains. Meprin β is an alternative β-secretase upregulated in brains of AD patients and capable of generating N-terminally truncated Aβ2-x peptides. Therefore, we aimed to generate a mouse model for the production of so far underestimated Aβ2-x peptides by conditionally overexpressing meprin β in astrocytes. We chose astrocytes as meprin β was detected in this cell type in close proximity to Aβ plaques in AD patients’ brains. The meprin β-overexpressing mice showed elevated amyloidogenic APP processing detected with a newly generated neo-epitope-specific antibody. Furthermore, we observed elevated Aβ production from endogenous APP as well as AD-related behavior changes (hyperlocomotion and deficits in spatial memory). The novel mouse model as well as the established tools and methods will be helpful to further characterize APP cleavage and the impact of different Aβ species in future studies.

**Supplementary Information:**

The online version contains supplementary material available at 10.1007/s00018-024-05139-w.

## Background

Alzheimer’s disease (AD) is the most common type of dementia [1] and the seventh-leading cause of death in the US in 2021 [2]. However, apart from two antibodies that recently received accelerated approval by the U.S. Federal Drug Administration (FDA) (https://www.fda.gov/drugs/news-events-human-drugs/fdas-decision-approve-new-treatment-alzheimers-disease, https://www.fda.gov/news-events/press-announcements/fda-grants-accelerated-approval-alzheimers-disease-treatment), the medication for patients is restricted to symptomatic treatments [3]. According to the amyloid cascade hypothesis, AD development is caused and driven by an increased production of extracellular neurotoxic amyloid-β (Aβ) peptides in the brain followed by hyperphosphorylation and intracellular aggregation of tau [4]. Aβ peptides, consisting of around 40 amino acids, are prone to aggregate [5]. As a result, diffusible oligomers are formed, which exhibit strong neurotoxic properties [6–8]. In later stages, these Aβ oligomers further aggregate into fibrils and then form large deposits in the brain, which are commonly referred to as amyloid plaques [9]. Aβ peptides are released upon proteolytic processing of the transmembrane amyloid precursor protein (APP) by so-called β- und γ-secretases, cleaving within the ectodomain and intramembranous, respectively. These cleavage events are referred to as the amyloidogenic pathway resulting in the release of a soluble APP fragment-β (sAPPβ) and Aβ [10]. In a competing non-amyloidogenic pathway, Aβ production can be prevented by cleavage of APP within the Aβ sequence by so-called α-secretases, thereby generating a soluble APP fragment-α (sAPPα) [11]. The first identified β-secretase was β-site APP cleaving enzyme 1 (BACE1) [12] as it is capable of cleaving APP between M671 and D672 (using the numbering of APP770 isoform) [13] and its protein expression is upregulated in AD patients’ brains [14, 15]. Therefore, D672 was considered as position 1 of Aβ [13], whereas the C-terminus of Aβ depends on the exact cleavage site of the γ-secretase complex. Particularly Aβ1-42, which exhibits strong neurotoxic properties, was identified in amyloid aggregates in AD brains, which led to the assumption that BACE1 is the major β-secretase and one of the most promising targets for therapeutic interventions [16–18]. However, all attempts to therapeutically inhibit BACE1 have so far been unsuccessful and have, therefore, been discontinued [19].

Detailed characterization of Aβ species occurring in AD patient brains revealed not only elevated Aβ1-x, but also different N-terminally truncated Aβ species such as Aβ2-42, which cannot be assigned to BACE1 activity [20]. Moreover, a critical view on widely used genetically modified AD mouse models to study amyloidogenesis demonstrated that in almost every model, the human Swedish APP (APPswe) variant is used [21]. APPswe contains two mutations proximal to the β-secretase site [22], which lead to dominant cleavage by BACE1 and thus strong Aβ1-x accumulation [23], whereas Aβ2-x peptides are absent in these mice [24]. Therefore, the role of BACE1 as the predominant and clinically most important β-secretase is currently reconsidered and the relevance of alternative β-secretases is evaluated in recent studies [21, 25–27]. Hence, the generation of novel APPswe-independent AD mouse models is of interest.

The metalloprotease meprin β was identified as an alternative β-secretase, which is upregulated in AD patients’ brains and capable of generating N-terminal truncated Aβ2-x peptides [28–30]. These peptides show a strong potential to aggregate [31] and are highly elevated in AD patients’ brains [20]. The possible pathological relevance of meprin β for Aβ production was demonstrated in a recent study, which shows that the knock-out of meprin β in a mouse model lacking the APPswe mutations and only harboring the further C-terminal located APP London (APPlon) mutation diminishes Aβ levels and deposition in the mouse brain and rescues cognitive deficits [30]. Of note, APPlon mice contain the mutation V717I proximal to the γ-secretase, whereas the β-secretase remains unaltered.

Since meprin β expression is upregulated in AD patients’ brains [28–30], we aimed to generate a meprin β knock-in mouse conditionally overexpressing meprin β in the brain to achieve pathological expression levels of meprin β. It has already been shown that meprin β is expressed in astrocytes and in close proximity to Aβ plaques in AD patients’ brains [29]. Moreover, the investigation of the Aβ production by primary brain cells revealed that astrocytes generate higher levels of Aβ2-42 than neurons and microglia [32]. Therefore, we generated meprin β-knock-in mice overexpressing murine meprin β specifically in astrocytes under the control of the GFAP promoter. These new mice are characterized in this study with respect to APP cleavage, Aβ release and deposition as well as their behavior to evaluate whether meprin β overexpression in astrocytes leads to an AD-like phenotype.

## Methods

### Cell culture, transfection, and lysis

HEK 293T cells were maintained at 37 °C under an atmosphere of 5% CO_2_ in Dulbecco’s modified Eagles medium (DMEM; Thermo Fisher Scientific) supplemented with 10% fetal bovine serum (FBS; Thermo Fisher Scientific). Transfection with plasmid-DNA, pre-mixed with polyethylenimine (PEI) (1:3) in serum-free medium was performed at 80–90% cell confluence. Plasmid DNA with mouse ADAM10, human and mouse APP695, human and mouse meprin β wt, a soluble human meprin β variant with stop codon that terminates translation N-terminal of the transmembrane domain, and pcDNA3.1 as empty vector control in different combinations were added together with transfection reagent to the cell culture medium. After 24 h, the cell medium was changed to serum-free DMEM. For SDS-PAGE and western blot analyses, harvested cells were washed with phosphate-buffered saline (PBS) and then incubated in lysis buffer (with freshly added cOmplete protease inhibitor cocktail (Roche), 1% (v/v) Triton X-100 in PBS (pH 7,4)) for 30 min at 4 °C. Lysates were centrifuged at 15,000×*g* at 4 °C and pelleted cell debris was discarded. The protein concentration in the lysates was determined using the BCA protein assay kit (Thermo Fisher Scientific) according to manufacturer’s instructions.

### Generation of conditioned supernatant containing shed and soluble meprin β

To generate conditioned supernatant containing shed meprin β, HEK cells were transfected with murine ADAM10 and human meprin β. To obtain conditioned supernatant with soluble meprin β, HEK cells were transfected with a meprin β variant containing a stop codon that terminates translation N-terminal of the transmembrane domain. After 24 h, the cell medium was changed to serum-free DMEM for 24 h. Afterward, the supernatant was removed, ultracentrifuged at 100,000×*g* for 1 h and treated with 5 µg/ml trypsin for 15 min to activate meprin β. Subsequently, trypsin was inhibited with 10 µg/ml ovomucoid for 15 min before applying the conditioned supernatant.

### Generation of a sAPPβ + Asp antibody

The sAPPβ + Asp antibody was generated by Pineda antibody service (Berlin, Germany). In brief, rabbits were immunized with a peptide consisting of the last five amino acids of the sAPPβ + Asp C-terminus with a GC linker (NH2-CGEVKMD-COOH) coupled to an immunogenic carrier protein. After 3 months, the antiserum was extracted and the IgG fraction was purified.

### SDS-PAGE and western blot analysis

Mouse brain homogenates, peptides, cell/OBSC lysates or culture supernatants were incubated for 10 min at 95 °C with sample buffer (50 mM Tris–HCl (pH 6.8), 2% (w/v) sodium dodecyl sulphate (SDS), 0.1% bromophenol blue (Merck), 10% (v/v) glycerol, 30 mg dithiothreitol (DTT)). The protein separation was performed by SDS-PAGE (120 V, 90 min) using the Mini-PROTEAN® system (Bio-Rad) or the Nu-Page system (Thermo Fisher Scientific). Afterward, proteins were visualized with Coomassie staining or it was continued conducting western blotting. The latter was accomplished with a tank-blot system (Bio-Rad) and protein transfer was onto PVDF membranes (0.8 A, 2 h, 4 °C). Afterward, the membranes were blocked with 5% milk (w/v) in TBS for 1 h at room temperature. The primary antibodies against meprin β (polyclonal antibody, generated against a peptide from the MAM domain (Pineda)), sAPPβ + Asp (polyclonal antibody, generated against the neo-C-terminus of sAPPβ, when APP is cleaved between D672 and A6273), sAPPβ (poly8134, Biolegend), HA-tag (6E2, Cell Signaling), HA-tag (C29F4, Cell Signaling) Notch-1 (ab27526, abcam), APP (poly158058, Biolegend) for the detection of murine Aβ and sAPPα, APP (CT15, described before in [33]) for the detection of APP in cell lysates and membrane fractions, APP (6E10, Biolegend) for the detection of human sAPPα, nicastrin (N1660, Sigma Aldrich), APP (22C11, eBioscience) for the detection of N-APP20, sez6 (14E5, kindly provided by Stefan Lichtenthaler, DZNE Munich, Germany [34]), GAPDH (14C10, Cell Signaling), Notch-1 (ab27526, abcam) and PSEN1 (7H8) were, unless not stated differently, incubated using a dilution of 1:1000 with the membrane over night at 4 °C. Horseradish peroxidase-conjugated secondary antibodies (Jackson ImmunoResearch) were diluted in TBS-T (TBS with 0.1% (v/v) Tween20) and incubated with the membranes for 1 h at room temperature. The chemiluminescence signal was detected in the Intelligent Dark Box (Fujifilm) or Amersham ImageQuant™ 800 (Cytiva) using the Super Signal® West Pico/Femto Kits (Thermo Fisher Scientific) according to manufacturer’s instructions. Western blot signals were quantified with ImageJ.

### sAPPβ-based peptides

Peptides representing the C-terminus of different sAPPβ species (Genosphere Biotechnologies) consisted of the following amino acids:

wt_sAPPβ + Asp: _626_ADSVPANTENEVEPVDARPAADRGLTTRPGSGLTNIKTEEISEVKMD_672_,

wt_sAPPβ: _626_ADSVPANTENEVEPVDARPAADRGLTTRPGSGLTNIKTEEISEVKM_671_, swe_sAPPβ + Asp: _626_ADSVPANTENEVEPVDARPAADRGLTTRPGSGLTNIKTEEISEV**NL**D_672_,

swe_sAPPβ: _626_ADSVPANTENEVEPVDARPAADRGLTTRPGSGLTNIKTEEISEV**NL**_671_.

### Aβ enzyme-linked immunosorbent assay (ELISA)

To quantify various Aβ species independent of the N-terminus, the Aβx-40 ELISA (LEGEND MAX™ β-Amyloid x-40 ELISA Kit with pre-coated plate; Biolegend) and Aβx-42 ELISA (LEGEND MAX™ β-Amyloid x-42 ELISA Kit with pre-coated plate; Biolegend) were used according to the manufacturer’s instructions diluting the samples 1:10. After the distribution of both ELISA Kits was discontinued by Biolegend, a comparable Aβx-40 and Aβx-42 sandwich ELISA was developed. For this purpose, 1 µg/ml anti-β-Amyloid, 1–40 (Clone: 11A50-B10; Biolegend) or 1 µg/ml anti-β-Amyloid, 1–42 (Clone: 12F4; Biolegend) diluted in PBS was used as coating antibody and transferred to a Nunc MaxiSorp™ Flat-Bottom Plate (Thermo Fisher Scientific) and incubated overnight at 4 °C. Afterward, the wells were washed three times with 300 µl TBS buffer and subsequently incubated with ELISA blocking buffer (1% (w/v) BSA in TBS) for 2 h at room temperature. Following another washing step, the samples were applied with a dilution of 1:10 together with 0.5 µg/ml of the detection antibody APP (4G8)-HRP (Biolegend) in a total volume of 100 µl ELISA incubation buffer (0.1% (w/v) BSA in TBS). After incubation overnight at 4 °C, the wells were washed five times with 300 µl TBS. The color development was achieved applying 100 µl tetramethylbenzidine (TMB) substrate (R&D) for 5–10 min. The reaction was stopped with 50 µl of 1 M sulfuric acid. The absorption was measured at 450 nm and quantified in comparison with Aβ2-40 or Aβ2-42 standards.

### Experimental animals

Mice were maintained under a 12-h light/12-h dark cycle with access to water and standard mouse diet ad libitum in individually ventilated cages (IVCs) in accordance with the ethical standards set by the National Animal Care Committee of Germany. All animal protocols were approved by the Central Animal Facility of the University of Kiel, Germany.

### Generation of mice overexpressing meprin β in astrocytes (*GFAP*^*Cre*+*/−*^*;Rosa26*^*Mep1b−HA*^)

Meprin β knock-in mice (Rosa26^Mep1b−HA^) were generated as described in [35]. To achieve meprin β overexpression in astrocytes, Rosa26^Mep1b−HA^ mice were crossed with mice expressing Cre recombinase under the control of the glial fibrillary acidic protein (GFAP) promoter, specifically active in astrocytes, which were described before [36]. *Rosa26*^*Mep1b−HA*^ mice, which were heterozygous for GFAP-dependent Cre, are termed as *GFAP*^*Cre*+/−^*;Rosa26*^*Mep1b−HA*^ mice (or briefly *GFAP*^*Cre*+/−^). The respective Cre-negative control animals are referred to as *GFAP*^*Cre−/−*^*;Rosa26*^*Mep1b−HA*^ (or briefly *GFAP*^*Cre−/−*^).

### Generation of mouse brain lysates and membrane/soluble fractions

Mice were sacrificed by cervical dislocation in accordance with the Guide for the Care and Use of Laboratory Animals (German Animal Welfare Act on Protection of Animals) and brains were isolated. For whole brain lysates, the brains were homogenized in triton lysis buffer (1% (v/v) triton X-100 (Roth), cOmplete protease inhibitor cocktail (Roche) (EDTA-free inhibitor cocktail was used, whenever a meprin β activity assay was applied) using the Precellys® 24 (VWR) for three cycles at 3000 rpm. The homogenates were incubated for 1 h at 4 °C. The debris was removed by centrifugation for 15 min at 16,000 × g and 4 °C. The membrane and soluble fractions were generated homogenizing mouse brains in homogenization buffer (2 mM Tris–HCl (pH 7.4), 250 mM sucrose, 0.5 mM EDTA, 0.5 mM EGTA, cOmplete protease inhibitor cocktail (Roche)) using a Douncer tissue homogenizer. The homogenates were centrifuged at 100,000 × g for 1 h. The supernatant was used as soluble fraction. The pellet was homogenized in RIPA lysis buffer (200 mM Tris–HCl (pH 7.5), 150 mM NaCl, 1 mM disodium EDTA, 1% (v/v) NP-40, 1% (w/v) sodiumdeoxycholate, 2.5 mM sodium pyrophosphate, cOmplete protease inhibitor cocktail (Roche)) using the Precellys® 24 (VWR) for three cycles at 3000 rpm and 4 °C. The homogenates were centrifuged at 100,000 × g for 1 h. The supernatant was used as membrane fraction. The protein concentration of all lysates was determined using Pierce™ BCA Protein Assay Kit (Thermo Fisher Scientific) according to the manufacturer´s instructions.

### Immunohistochemistry (IHC)

Mouse brains were dissected from 1-year-old *GFAP*^*Cre*+/−^*;Rosa26*^*Mep1b−HA*^ and *GFAP*^*Cre−/−*^*;Rosa26*^*Mep1b−HA*^ mice and fixed in 4% paraformaldehyde (PFA) in PBS overnight. The samples were dehydrated using ethanol as well as xylene-based solutions and afterward, the tissues were embedded in low-melting-point paraffin according to standard laboratory procedures. Three-µm-thick sections were generated, deparaffinated and immunostained using the Ventana Benchmark XT machine (Ventana, Roche Diagnostics). For this purpose, the sections were boiled in CC1 buffer (Ventana, Roche Diagnostics) for antigen retrieval according to the manufacturer’s instructions. The sections were incubated with primary antibodies detecting GFAP (1:200, M0761, Dako), Iba-1 (1:500, 019-19741, Wako), NeuN (1:50, MAB377, Merck), β-Amyloid (1:100, 4G8, Biolegend), and HA-tag (1:100, C29F4, Cell Signaling) in IHC incubation buffer for 1 h at 4 °C. The secondary antibody was applied using the anti-rabbit histofine Simple Stain MAX PO Universal immunoperoxidase polymer or Mouse Stain Kit (both from Nichirei) according to the manufacturer’s instructions. The detection was conducted with the Ultra View Universal DAB Detection Kit (Ventana, Roche Diagnostics) according to the standard settings of the machine. Sections from *GFAP*^*Cre*+/−^*;Rosa26*^*Mep1b−HA*^ and *GFAP*^*Cre−/−*^*;Rosa26*^*Mep1b−HA*^ were stained in one run to achieve identical conditions. The counterstaining was also conducted by the machine according to the standard settings. Pictures were taken with the EP50 (Leica).

### Fluorogenic peptide-based activity assay

The activity of meprin β in lysates of mouse brains or OBSCs was measured with a well-established and highly specific quenched fluorogenic peptide substrate ((mca)-EDEDED-(K-e-dnp); mca: 7-methyloxycoumarin-4-yl, dnp: dinitrophenyl) [37]. For this purpose, 300 µg of lysate was mixed with 50 µM quenched fluorogenic peptide substrate transferred into 96-well plates in a total volume of 100 µl PBS. If indicated in the respective figure legend, the samples were pre-incubated with 50 µM actinonin for 15 min at 37 °C before adding the peptide substrate. The measurement was performed at 37 °C for 2 h. Every 30 s, the fluorescence at λ_ex_ = 320 nm and λ_em_ = 405 nm was monitored. Relative fluorescent units (RFUs) were normalized to the respective control samples only containing lysis buffer and peptide substrate.

### Generation, cultivation, and harvest/lysis of organotypic brain slice cultures (OBSCs)

To generate OBSCs, mice were sacrificed by cervical dislocation in accordance with the Guide for the Care and Use of Laboratory Animals (German Animal Welfare Act on Protection of Animals). The head was disinfected in 70% (v/v) ethanol for 1 min and the brain was removed. Afterward, the brain was sagittally cut in the middle of one hemisphere with a razorblade. The cut side was subsequently glued on the specimen plate of a vibratome (VT1200S, Leica) proximal to a fixed 1 cm^3^ block of 2% (w/v) agarose. The specimen plate was set into its fixture within the vibratome filled with OBSC cutting medium (100 U/ml penicillin, 100 µg/ml streptomycin; 10 mM HEPES in HBSS). Using the vibratome, 180 µm (for immunofluorescence analysis) or 250 µm (for the generation of lysates) sagittal OBSCs were generated with a speed of 0.03 mm/s and an amplitude of 3 mm. The OBSCs were transferred on a membrane insert with a pore size of 0.4 µm (Greiner AG) in a six-well plate and cultivated in 1.8 ml OBSC medium (45% (v/v) MEM, 25% (v/v) horse serum, 19% (v/v) HBSS, 6.5 mg/ml D-( +)-glucose, 2 mM glutamine, 25 mM HEPES, 100 U/ml penicillin, 100 μg/ml streptomycin) (two slices per membrane insert) for 20 days. The brain slices were incubated at 37 °C in a 5% CO_2_ humidified incubator, changing half of the medium every 3 days. To avoid meprin β inhibition by serum components, the OBSCs culture medium was substituted by serum-free OBSC medium for 24 h before harvest. For tissue lysis, OBSCs were transferred into reaction tubes and incubated for 1 h at 4 °C in triton lysis buffer (EDTA-free lysis buffer was used, whenever a meprin β activity assay was applied). The debris was removed by centrifugation for 15 min at 16,000 × g and 4 °C. The protein concentration of all lysates was determined using Pierce™ BCA Protein Assay Kit (Thermo Fisher Scientific) according to the manufacturer´s instructions.

### MTT viability assay of OBSCs

After 20 days in culture, the medium of OBSCs was replaced with serum-free brain slice culture medium containing 0.5 mg/ml 3-[4,5-dimethylthiazole-2-yl]-2,5-diphenyltetrazolium bromide (MTT). Control slices were fixed with 4% PFA overnight at 4 °C and washed once with PBS prior to the incubation in MTT-containing medium. After 1 h at 37 °C, photographs of the slices were taken. Then the OBSCs were lysed in 10% (w/v) SDS for 24 h and the absorption was measured at 562 nm and 690 nm. The viability was calculated by subtracting A_690_ from A_562_.

### Immunofluorescence (IF) microscopy

For immunofluorescence staining of OBSCs, 180-µm thick slices were cultivated for 14 days. The medium was removed and the OBSCs were fixed using 4% (m/v) PFA in PBS at 4 °C overnight. Afterward, the OBSCs were cut out with a few millimeters of surrounding membrane and transferred to a 24-well plate. The OBSCs were washed three times with PBS for 5 min and incubated with OBSC blocking and permeabilizing solution (10% (v/v) FBS, 0.5% (v/v) Triton X-100 in PBS) for 5 h at room temperature. Antibodies against GFAP (ASTRO6, 1:200, Thermo Fisher Scientific) and HA-tag (C29F4, 1:1000, Cell Signaling) were diluted in PBS and incubated with the OBSCs overnight at 4 °C. After four washing steps for 20 min with PBS, the OBSCs were incubated with Donkey anti-Mouse IgG Alexa Fluor® 488 (Thermo Fisher Scientific) and Donkey anti-Rabbit IgG Alexa Fluor® 594 (Thermo Fisher Scientific) diluted 1:300 in PBS for 5 h at room temperature. After another washing step with PBS (four times for 20 min), the slices were incubated with 1 µg/ml DAPI in PBS for 5 min at room temperature followed by another three washing steps for 5 min. Afterward, the OBSCs were mounted using fluorescence mounting medium (Dako) and analyzed with a confocal microscope (FV1000, Olympus).

### Behavioral tests

The behavioral tests were conducted testing seven to eight 1.5-year-old mice per group. The mice were recorded using an overhead CCTV camera and video tracking software Ethovision 3.1 Software (Noldus).

#### Open field

Motor activity and explorative behavior were analyzed in a square white Perspex arena (40 × 40 cm). The mice were placed in the arena and tracked for 10 min. The arena was cleaned thoroughly between animals to eliminate odor cues. Speed, distance, and time spent in periphery or center were measured.

#### Elevated plus maze

Mice were tested for anxiety behavior in an elevated plus maze. The plus-shaped maze consisted of two open and two with walls enclosed arms. The mice were placed in the center square. Moving paths of the mice were recorded for 10 min per mouse. The maze was cleaned thoroughly between animals to eliminate odor cues. Anxiety was quantified by comparing the time, which the mice spent in the open and enclosed arms, respectively.

#### Y maze

As Y maze, a white Perspex arena consisting of three equally sized arms in a “Y” configuration was used. The mice were placed in the end of one arm facing to the wall and were allowed to explore the arena for 10 min. A complete alternation was achieved when the animal entered three different arms in a row. The alternation was calculated by dividing correct sequences by the number of total entries minus two. The maze was cleaned thoroughly between animals to eliminate odor cues.

#### Spatial object recognition

The spatial object recognition was conducted in a round-shaped arena (diameter: 46 cm) with walls. On the first day, habituation started with mice placed individually in the arena without relevant objects and allowed to explore for 5 min. After a 5-min inter-trial interval, this procedure was repeated with three different cues (black and white shapes) attached on the internal wall of the maze, to provide spatial references. On the 2nd day, two objects with different colors and shapes were placed in the arena. After 5-min exploration and 5-min inter-trial interval, one of the objects was displaced 12 cm away from its original position. Mice were allowed to explore, one at the time, again for 5 min. For half of the mice of each group, stationary and moved objects were swapped, to avoid bias due to object preferences. The maze was cleaned thoroughly between animals to eliminate odor cues.

Moving path of the mice and duration were recorded. Mice with a total exploration time below 1 s were excluded, leaving five mice in the *GFAP*^*Cre−/−*^ and eight in the *GFAP*^*Cre*+/−^ group. Exploration time and frequency of stationary and moved objects, total exploration time, and recognition indices (RI) were analyzed. The formulas RI_moved_ = Tm/(Ts + Tm)*100 and RI_stationary_ = Ts/(Ts + Tm)*100 were used for recognition indices of moved and stationary object respectively, where Tm stands for time with moved object and Ts stands for time with stationary object [38].

#### Open field water maze

A pool with diameter 120 cm was filled with clear water, 30 cm deep (20 °C). Four black and white symbols around the maze provided extra-maze cues. The mice were released facing the wall into one of the four quadrants, called N, E, S or W, for each trial (order changed every day). Groups of 4 to 6 mice received four training trials on a single day with a 15-min inter-trial interval. Mice had 90 s to find a fixed submerged platform (6.0 cm diameter), located between the E and S or the N and W quadrants according to the subgroup, to exclude preference biases. The platform was submerged approximately 1 cm below the surface of the water. Mice had to reach and sit on the platform for at least 2 s to complete the task. Following both unsuccessful and successful trials, they were, respectively, placed or left on the platform for 15 s before being returned to their home cage. In this way, they had a chance to memorize the right location. Significant dropping of task latency and path length to reach the platform indicated effective learning and the end of the training. Retention for the platform location was tested with a probe trial 15 min after the last training trial on day 9. During probe trials, the platform was removed and the mice allowed to swim for 75 s. Swimming paths of the mice and latencies were recorded.

### Statistical analysis and illustrations

All statistical analyses were performed with the GraphPad Prism 7 software. The particular test, which was conducted, is always stated in the respective figure descriptions (ns: *p* > 0.05; *: *p* ≤ 0.05; **: *p* ≤ 0.01; ***: *p* ≤ 0.001). The figures were created with Microsoft PowerPoint and BioRender.com.

## Results

### Amyloidogenic processing of endogenous APP by meprin β can be detected with a neo-epitope-specific sAPPβ antibody

**Fig. 1:**
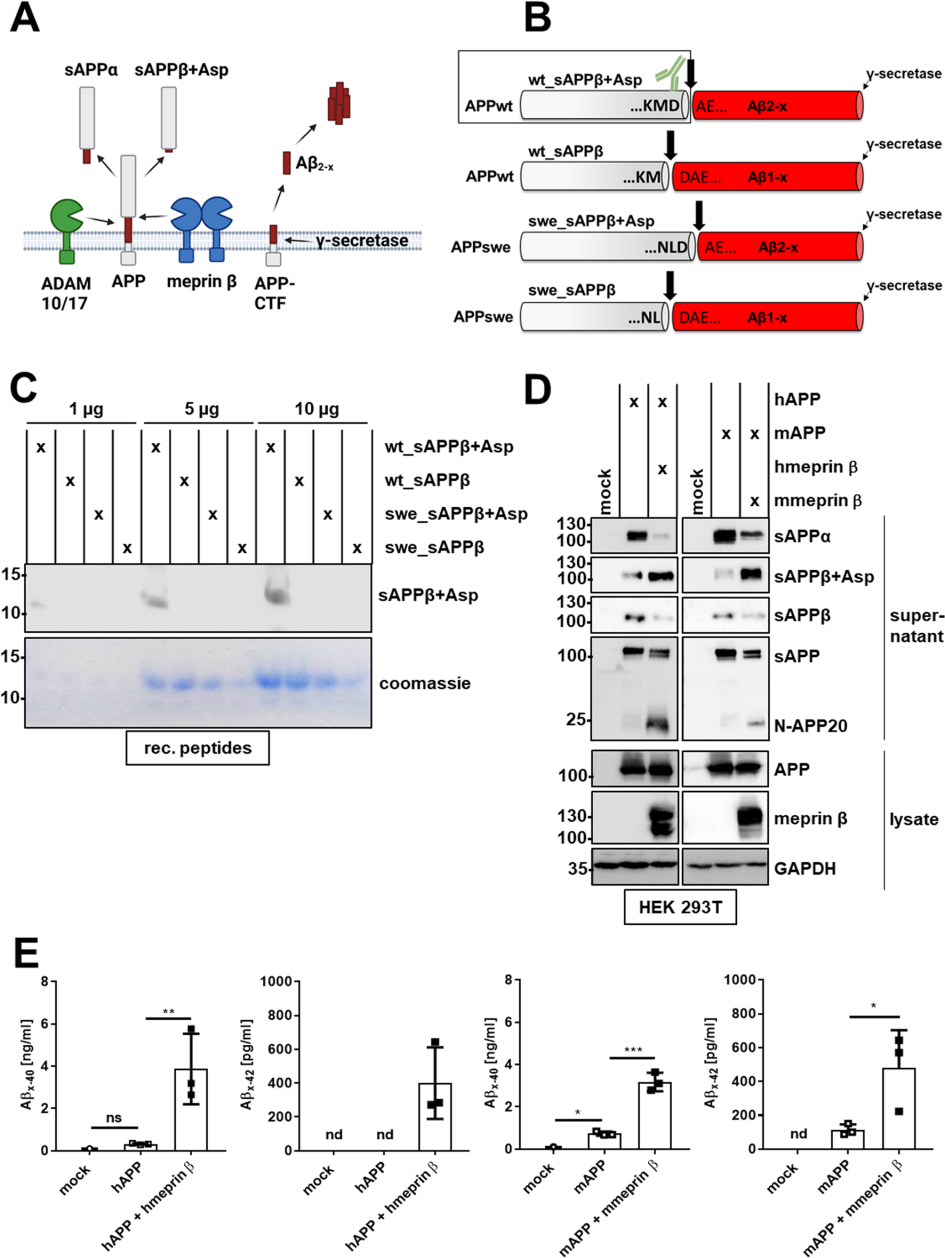
A newly generated neo-epitope-specific sAPPβ + Asp antibody detects human and mouse soluble APP processed by meprin β. **A** Meprin β is expressed at the cell surface and is capable of cleaving APP at the β-secretase site releasing the soluble APP fragment sAPPβ + Asp. The remaining C-terminal fragment (APP-CTF) can be further processed by the γ-secretase. As a result, the N-terminally truncated Aβ peptide Aβ2-x is generated. A disintegrin and metalloprotease 10 and 17 (ADAM10/17) act as α-secretases, releasing the soluble APP fragment sAPPα and thereby preventing Aβ formation. **B** The peptides analyzed in **C** are depicted. The peptides comprise the 46 or 47 C-terminal amino acids of the following sAPPβ species. Upon cleavage between D672 and A673, N-terminally truncated Aβ2-x and C-terminally extended sAPPβ (wt_sAPPβ + Asp) are generated from wild-type APP (APPwt). When APPwt is cleaved between M671 and D672, Aβ1-x and conventional sAPPβ (wt_sAPPβ) is generated. Both sAPPβ species carrying the Swedish APP (APPswe) mutations (swe_sAPPβ + Asp and swe_sAPPβ) are also depicted. Wt_sAPPβ + Asp, which is specifically detected by the neo-epitope-specific antibody (depicted in green), is highlighted in a black box. **C** 1–10 µg of the synthesized peptides schematically depicted in **B** were analyzed using SDS-PAGE with consecutive Coomassie brilliant blue staining and western blotting using the neo-epitope-specific sAPPβ + Asp antibody. **D** HEK cells were transfected with either human APP (hAPP) and human meprin β (hmeprin β) or murine APP (mAPP) and murine meprin β (mmeprin β) as indicated. Cell lysates and supernatants were analyzed by SDS-PAGE and western blot. **E** The cell culture supernatants from **D** were analyzed using the Aβx-40 and Aβx-42 ELISA (*n* = 3). The significance was determined by one-way ANOVA (ns: *p* > 0.05; *p ≤ 0.05; **p ≤ 0.01; ***p ≤ 0.001). nd—below the detection limit of the ELISA

To mimic meprin β upregulation as observed in AD patients’ brains [28–30], we aimed to develop a mouse model, in which meprin β is overexpressed in astrocytes. Membrane-bound meprin β is capable of cleaving APP at the β-secretase site one amino acid shifted toward the C-terminus compared to the BACE1 cleavage site [28, 29] (Supplementary Fig. S1A, B). Of note, APP can also be cleaved by meprin β at its N-terminus releasing the so-called N-APP20 fragment [39]. It can be generated by membrane-bound meprin β as well as the soluble meprin β ectodomain. The release of N-APP20 is not related to AD as it is not neurotoxic; however, it is a sensitive tool to specifically detect meprin β activity. As a result, N-terminally truncated Aβ2-x peptides, which are described to be mainly produced by astrocytes, as well as C-terminally prolonged sAPPβ fragments (referred to as sAPPβ + Asp) are released (Fig. 1A). To specifically detect amyloidogenic APP processing by meprin β, we generated a neo-epitope-specific polyclonal antibody against sAPPβ + Asp. The resulting antibody is highly specific for sAPPβ + Asp and neither detects wt BACE1-generated sAPPβ nor the sAPPβ or sAPPβ + Asp species derived from the widely used APPswe variant with two mutations N-terminal to the β-secretase site (Fig. 1B). Antibody specificity was validated with synthetic peptides representing the C-terminus (last five amino acids) of the different sAPPβ species (Fig. 1C) and in cell culture comparing APP cleavage by BACE1, generating Aβ1−x, and meprin β, producing Aβ2-x (Supplementary Figure S2). To evaluate whether amyloidogenic APP cleavage by meprin β might occur in the murine system (as observed previously for the human proteins [28, 29]), we transfected HEK293T cells with the human and murine forms of APP and meprin β. Each combination of murine and human proteins resulted in elevated amyloidogenic APP cleavage depicted by reduced levels of sAPPα and increased release of C-terminally extended sAPPβ + Asp and Aβ (Fig. 1D, E and Supplementary Figure S3A, B). Of note, both human and mouse sAPPβ + Asp can be detected with the neo-epitope-specific sAPPβ + Asp antibody. The conventional sAPPβ levels were detectable only after an extended exposure time. Following meprin β transfection, conventional sAPPβ levels were decreased, presumably due to meprin β competing with endogenously expressed β-secretases.

### Generation of a mouse line overexpressing meprin β in astrocytes

**Fig. 2:**
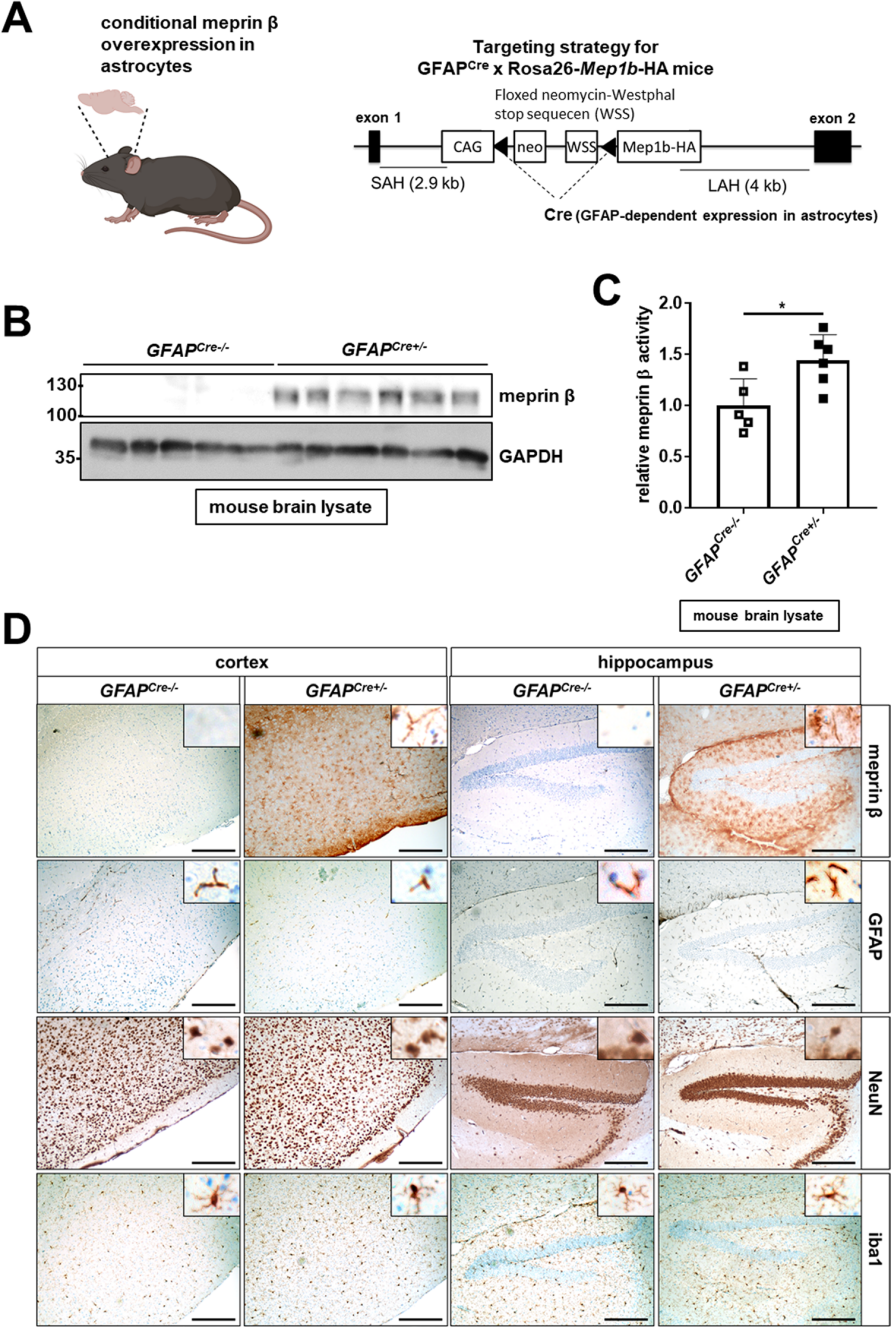
Generation of a mouse model with GFAP^Cre^-dependent meprin β overexpression in astrocytes. **A** Scheme of the generation of a mouse model overexpressing meprin β in astrocytes. *GFAP*^*Cre*+/−^*;Rosa26*^*Mep1b−HA*^ mice contain a gene coding for C-terminal HA-tagged murine meprin β (*Mep1b*-HA) under the control of a CAG promotor. Transcription is prevented by a floxed neomycin-Westphal stop sequence (WSS) cassette. Only after crossing with *GFAP*^*Cre*^ mice, Cre is expressed in astrocytes and cuts out the floxed neomycin-WSS cassette, thereby inducing meprin β overexpression. SAH = short arm of homology; LAH = long arm of homology. **B** Brains from 1-year-old *GFAP*^*Cre*^*;Rosa26*^*Mep1b−HA*^ (*GFAP*^*Cre*+/−^) and control mice (*GFAP*^*Cre−/−*^) were homogenized. Lysates were analyzed with SDS-PAGE and western blot. **C** Brains from 1-year-old *GFAP*^*Cre*^*;Rosa26*^*Mep1b−HA*^ mice and controls were homogenized. Meprin β activity in the lysates was measured with or without the addition of 50 µM actinonin using a quenched fluorogenic peptide (*n* = 5–6). The bar graphs display the slope of the linear gain of fluorescence within the first 30 min. The significance level was determined by two-way ANOVA (ns: *p* > 0.05; **: *p* ≤ 0.01).** D** Brain sections from 1-year-old *GFAP*^*Cre*^*;Rosa26*^*Mep1b HA*^ mice and controls were IHC stained. Pictures were taken with a 10 × objective. Higher magnification pictures in the respective upper right corner were taken using the 40 × objective. The scale bars indicate 200 µm

To generate meprin β-overexpressing mice, we used floxed knock-in mice (*Rosa26*^*Mep1b*−HA^) containing murine meprin β cDNA with a C-terminal HA-tag inserted into the Rosa26 locus. Between promoter and murine meprin β cDNA is a neomycin-Westphal stop sequence (WSS) cassette surrounded by loxP sites to prevent from transcription. These mice were crossed with *GFAP*^*Cre*^ mice, almost exclusively expressing Cre and inducing meprin β overexpression in astrocytes (Fig. 2A, B). The resulting mice were viable, fertile, and did not differ in body and brain weight compared to Cre-negative animals (Supplementary Figure S4A, B). In brain lysates of 1-year-old *GFAP*^*Cre*+/−^*;Rosa26*^*Mep1b−HA*^ mice, high levels of meprin β were detected (Fig. 2B), which is partly activated by endogenous activators as 30% increased meprin β activity was detected in a fluorogenic peptide-based cleavage assay using full brain lysates (Fig. 2C). The low activation rate might be due to the fact that known tryptic meprin β activators such as matriptase-2, kallikreins, and trypsin are hardly expressed in astrocytes [40]. The addition of the meprin inhibitor actinonin led to a reduction of meprin β activity in brain lysates of meprin β-overexpressing and control mice, equalizing their activity values. This indicates the inhibition of both endogenous and overexpressed meprin β. IHC analyses confirmed meprin β overexpression in the brain of these mice (Fig. 2D).

### Meprin β overexpression in astrocytes leads to excessive amyloidogenic APP processing and

#### Aβ generation

**Fig. 3:**
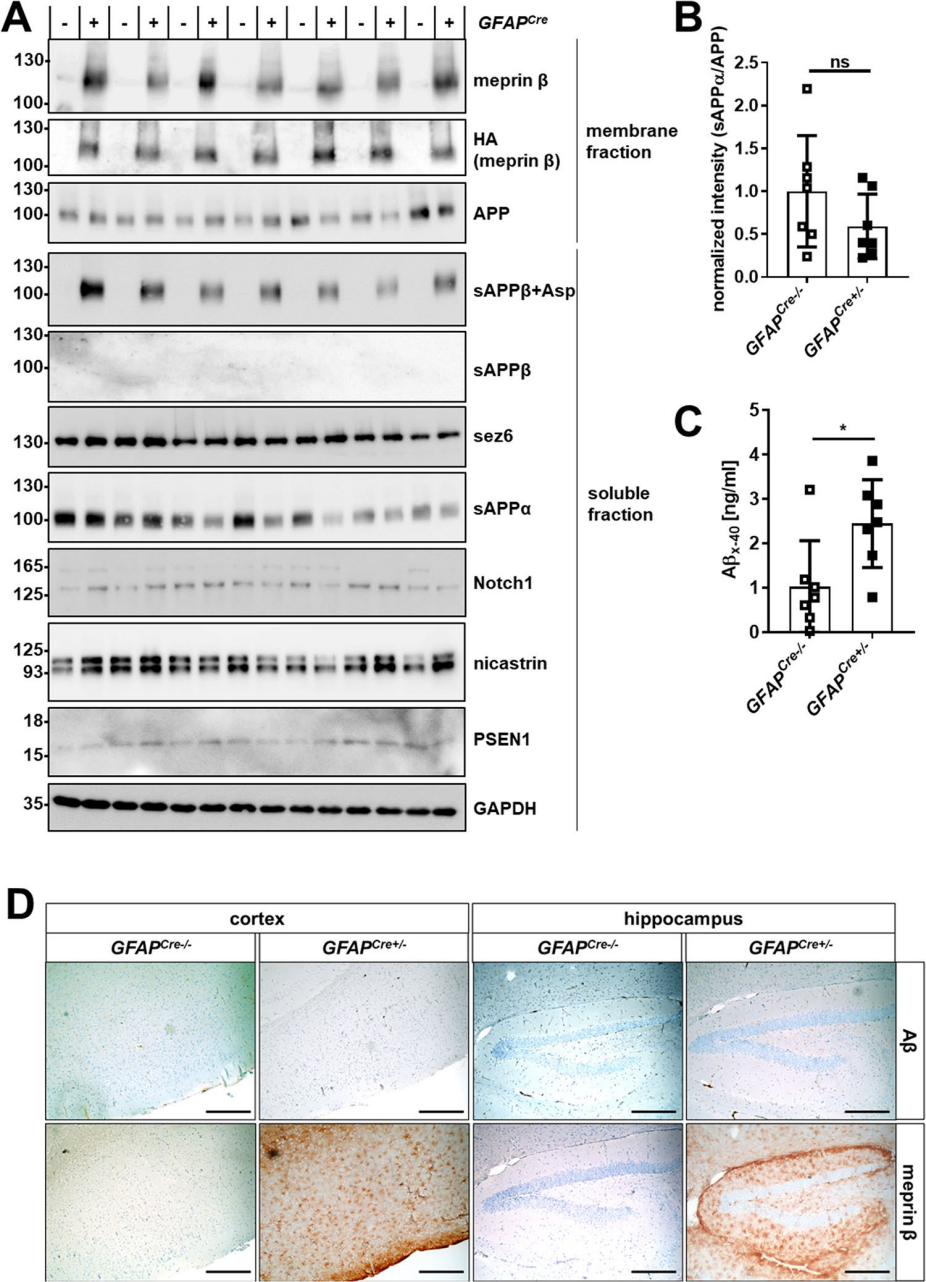
Meprin β overexpression in astrocytes leads to excessive amyloidogenic APP processing and Aβ generation. **A** Brains from 1.5-year-old *GFAP*^*Cre*^*;Rosa26*^*Mep1b−HA*^ ( +) and control mice ( −) were homogenized. Membrane and soluble fractions were generated and analyzed by SDS-PAGE and western blot. **B** The sAPPα signals of (A) were quantified and normalized to the APP signal (*n* = 7). The significance level was determined by *t* tests (ns: *p* > 0.05). **C** The mouse brain lysates of 1-year-old mice were analyzed using an Aβx-40 ELISA (*n* = 7). The significance level was determined by *t* tests (ns: *p* > 0.05; *: *p* ≤ 0.05). **D** Brain sections from 1-year-old *GFAP*^*Cre*^*;Rosa26*^*Mep1b−HA*^ mice (*GFAP*^*Cre*+/−^) and control mice (*GFAP*^*Cr*−/−^) were IHC stained. To show meprin β expression, the same IHC images as in Fig. 2D are depicted. The scale bars indicate 200 µm

To analyze the impact of astrocytic meprin β on amyloidogenic APP processing and Aβ release, membrane and soluble fractions of mouse brains were generated. Western blot analyses showed increased levels of sAPPβ + Asp in the soluble fraction, which correlates with the meprin β overexpression in membrane fraction detected with a meprin β-specific as well as an HA-tag antibody (Fig. 3A). However, conventional sAPPβ was not detectable in both meprin β-overexpressing and control mouse brain lysates. Of note, the signals in western blot of full-length APP as substrate, but also of nicastrin and PSEN1 as parts of the γ-secretase complex, were not altered upon meprin β overexpression. The same holds true for sez6, a substrate of the major β-secretase BACE1 and Notch-1, which is a substrate of the constitutive α-secretase ADAM10 (see Fig. 3A and Supplementary Figure S5A, B). This supports previously obtained results [31] that meprin β directly cleaves APP, and amyloidogenic APP processing does not occur through secondary cleavage events. It has been described that α- and β-secretases such as ADAM10 and meprin β compete for APP as a substrate and that upregulation of β-secretases diminishes sAPPα levels [41–43]. However, sAPPα levels were not significantly altered in the brains of *GFAP*^*Cre*+/−^*;Rosa26*^*Mep1b−HA*^ mice (Fig. 3B). Using an Aβx-40 ELISA, elevated Aβ levels were detected in meprin β-overexpressing mice, showing that amyloidogenic APP processing by meprin β results in Aβ formation (Fig. 3C). However, conducting Aβ IHC staining no plaque deposition was observed in respective mouse brains (Fig. 3D). This finding is in line with previous observations that aged wild-type mice without transgenic expression of human APP do not show plaque deposition [44].

### Organotypic brain slice cultures of meprin β-overexpressing mice show increased Aβ release

**Fig. 4:**
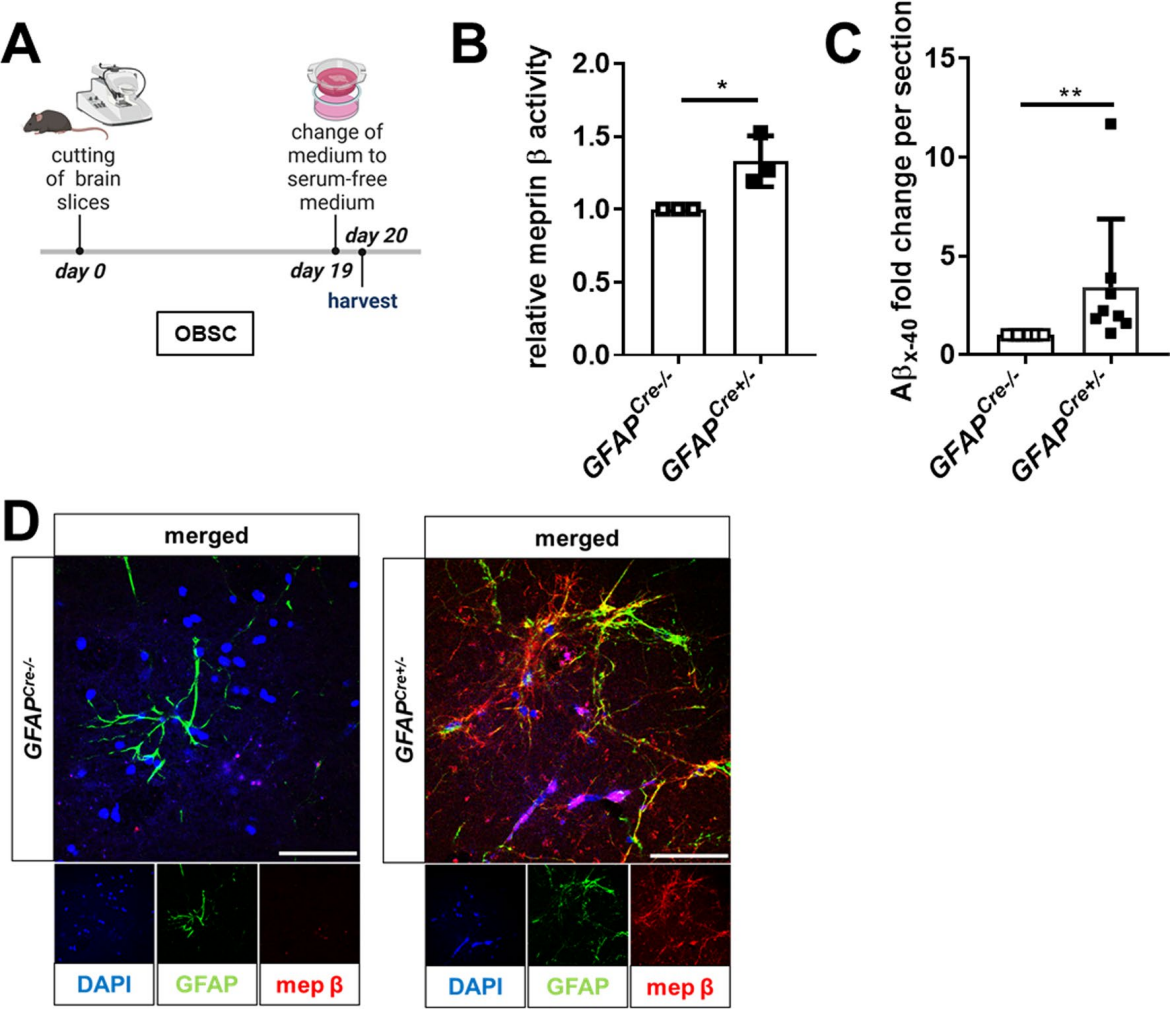
OBSCs from meprin β-overexpressing mice show elevated Aβ release. **A** Cultivation procedure for organotypic brain slices (OBSCs). Brains from *GFAP*^*Cre*^*;Rosa26*^*Mep1b−HA*^ mice (*GFAP*^*Cre*+/−^) and control mice (*GFAP*^*Cre−/−*^) were cut into 250-µm thick sections and cultivated for 20 days. On day 19, the medium of the OBSCs was changed to serum-free medium for 24 h. Then the OBSCs were harvested. **B** The OBSCs were lysed and meprin β activity was measured using a highly specific quenched fluorogenic peptide substrate (*n* = 3). The bar graphs display the slope of the linear gain of fluorescence within the first 30 min. The significance level was determined by *t* test (ns: *p* > 0.05; *: *p* ≤ 0.05). **C** Statistical analysis of normalized Aβx-40 levels measured in sequentially cut OBSCs (Supplementary Figure S4D) (*n* = 8). A Wilcoxon matched-pairs signed test was used to determine statistical significance (ns: *p* > 0.05; *: *p* ≤ 0.05; **: *p* ≤ 0.01). **D** Brains from *GFAP*^*Cre*^*;Rosa26*^*Mep1b−HA*^ mice and controls were cut into 180-µm sections and cultivated for 14 days. The OBSCs were subsequently IF stained as indicated. Confocal images are depicted. The scale bar indicates 50 µm

To further characterize the amyloidogenic APP processing by meprin β in an ex vivo model, we generated OBSCs, which were cultivated for 20 days. At day 19, the culture medium was changed to serum-free medium to avoid meprin β inhibition by serum components (Fig. 4A). Applying an MTT-based assay, the viability of 20-day-old OBSCs was validated (Supplementary Figure S6A, B). The overexpressed meprin β in OBSC lysates is partly activated by endogenous activators (Fig. 4B) as observed in the corresponding mouse brain lysates (Fig. 2C). Meprin β overexpression led to the release of Aβ into the supernatant of cultured OBSCs (Fig. 4C). Conducting immunofluorescence (IF) staining of 20-day-old OBSCs, we validated meprin β expression in astrocytes by co-localization with GFAP (Fig. 4D). To characterize the brain region, in which astrocytic meprin β is involved in Aβ production, we generated sequential slices of one brain hemisphere (Supplementary Figure S6C). Western blot analyses (for meprin β) and ELISA measurements (for released Aβ) revealed that, in the periphery of the brain hemisphere, both meprin β expression and Aβ production are lower than in the center of the hemisphere (Supplementary Figure S6D, E).

### Meprin β-overexpressing mice show enhanced locomotion and deficits in spatial object recognition

**Fig. 5:**
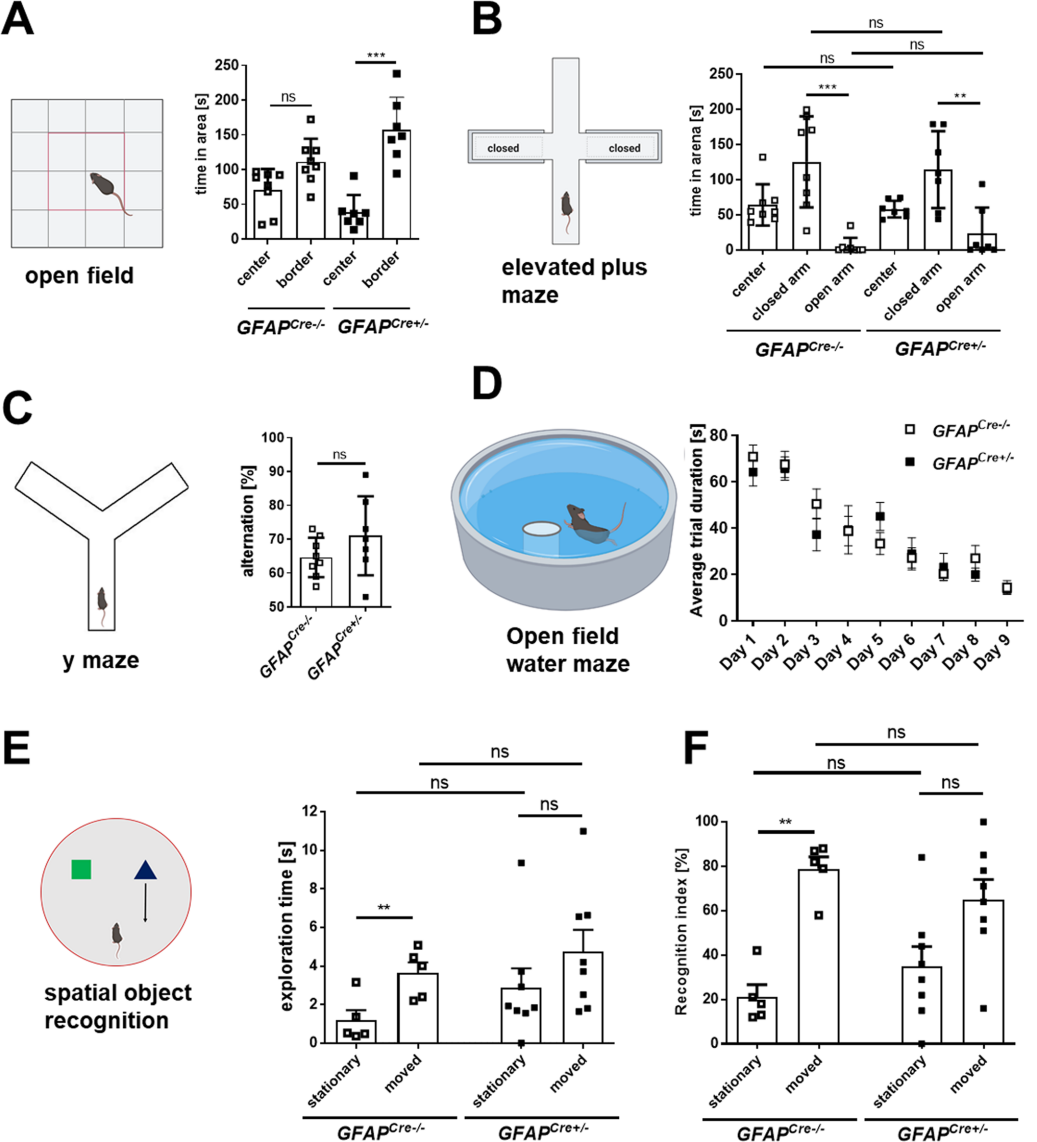
Meprin β overexpression in astrocytes leads to higher locomotion and deficits in spatial object recognition. **A** Open field test. The time mice spent in the center and border was acquired. **B** Elevated plus maze. Scheme of the apparatus, with two closed and two open arms. The time mice spent in the center, closed arm, and open arm was acquired. **C** Y maze. Spontaneous alteration of mice was quantified. **D** Open field water maze. The average latencies of four trials were plotted. **E** Spatial object recognition. The graph shows the exploration time, acquired tracking the nose in the object area, during a trial with one object located in the familiar position and one object displaced to a new location. **F** Spatial object recognition. The graph shows the recognition index of both groups, calculated by dividing the time spent exploring the stationary or the moved object by the total exploration time and then multiplying per 100 (%). The behavioral tests were conducted with *n* = 7–8 mice. The significance levels were determined by *t* tests except for **A**, **B**, and **D**, which were calculated using two-way ANOVA (ns: *p* > 0.05; *: *p* ≤ 0.05; **: *p* ≤ 0.01)

To investigate whether the generation of Aβ upon meprin β overexpression would induce behavioral changes, we subjected meprin β and control mice to behavioral tests. In the open field, both groups of mice spent more time in the border zone than the center. However, this difference was significant only for meprin β but not control mice (Fig. 5A, time in area, border vs center, *GFAP*^*Cre−/−*^ two-way ANOVA *p* = 0.1072 *n* = 8, *GFAP*^*Cre*+/−^ p < 0.001 *n* = 7). The difference in exploratory behavior could not be explained by increased anxiety, as both groups did not differ in the elevated plus maze (EPM), a sensitive test of anxiety levels (Fig. 5B, time in closed arms, two-way ANOVA *p* = 0.9943 *GFAP*^*Cre−/−*^ mean ± SEM 125.0 ± 22.85 s, *n* = 8, *GFAP*^*Cre*+/−^ mean ± SEM 114.0 ± 20.69 s, *n* = 7 – time in open arms, *t* test *p* = 0.9535 *GFAP*^*Cre−/−*^ mean ± SEM 5.005 ± 4.225 s, *n* = 8, *GFAP*^*Cre*+/−^ mean ± SEM 22.96 ± 14.02 s, *n* = 7). To probe for changes in mnemonic performance, we initially analyzed working memory in a spontaneous alternation task in a Y maze, which showed no significant differences between groups (Fig. 5C, spontaneous alternation, *t* test *p* = 0.1949 *GFAP*^*Cre−/−*^ mean ± SEM 64.63 ± 2.061%, *n* = 8, *GFAP*^*Cre*+*/−*^ mean ± SEM 71.00 ± 4.413%, *n* = 7). Similarly, meprin β-overexpressing mice showed no difference in incremental spatial reference memory as tested in the open field water maze. Both groups of mice learned the platform location as indicated by the reduction in latency over several days of training (*GFAP*^*Cre−/−*^ one-way ANOVA F (3.566, 24.96) = 19.06 p < 0.0001, *GFAP*^*Cre*+/−^ one-way ANOVA F (3.115, 18.69) = 9.392 *p* = 0.0005) (Fig. 5D) and did not differ significantly from each other (latency: two-way ANOVA F (1, 13) = 0.1430, *p* = 0.7114; path length: two-way ANOVA F (1, 13) = 0.6000, *p* = 0.4524; probe trial, time in platform quadrant: *GFAP*^*Cre−/−*^ vs *GFAP*^*Cre*+/−^* t* test *p* = 0.9043, *GFAP*^*Cre−/−*^ vs chance *t* test *p* = 0.0992, *n* = 8, *GFAP*^*Cre*+/−^ vs chance *t* test *p* = 0.0140, *n* = 7) (Figs. 5D, S7A-B).

However, when we tested for spatial short-term memory in a spatial object recognition paradigm, we observed that meprin β-overexpressing mice performed worse than control animals (Figs. 5E–F, S7C). Although meprin β-overexpressing mice did not significantly differ from control mice in overall exploration times (*t* test *p* = 0.1564), they failed to recognize the relocation of the object, suggesting impaired short-term spatial memory (exploration time, moved vs stationary object: *GFAP*^*Cre−/−*^* t* test *p* = 0.0074, *n* = 5, *GFAP*^*Cre*+/−^* t* test *p* = 0.2950 *n* = 8; recognition index, moved vs stationary object, *GFAP*^*Cre−/−*^* t* test *p* = 0.0062 *n* = 5, *GFAP*^*Cre*+/−^* t* test *p* = 0.1357 *n* = 8; exploration frequency, moved vs stationary object: *GFAP*^*Cre−/−*^* t* test *p* = 0.0086, *n* = 5, *GFAP*^*Cre*+/−^* t* test *p* = 0.1497 *n* = 8). (Figs. 5E–F, S7C). In addition to the deficit in spatial object recognition, we noticed that *GFAP*^*Cre*+/−^ mice displayed enhanced locomotion as indicated by increased running speed (Supplementary Figure S7D, open field: *t* test *p* = 0.0534, *GFAP*^*Cre−/−*^ mean ± SEM 21.82 ± 1.436 mm/s, *n* = 8, *GFAP*^*Cre*+/−^ mean ± SEM 26.59 ± 1.751 mm/s, *n* = 7; EPM: *t* test *p* = 0.0016,*GFAP*^*Cre−/−*^ mean ± SEM 36.03 ± 1.402 mm/s, *n* = 8, *GFAP*^*Cre*+/−^ mean ± SEM 51.60 ± 3.882 mm/s, *n* = 7; Y maze: *t* test *p* = 0.0373,*GFAP*^*Cre−/−*^ mean ± SEM 48.03 ± 3.472 mm/s, *n* = 8, *GFAP*^*Cre*+/−^ mean ± SEM 59.11 ± 3.207 mm/s, *n* = 7).

## Discussion

Meprin β is upregulated in AD patients’ brains and as an alternative β-secretase likely involved in the generation of pathological relevant Aβ peptides [26, 28–30]. A recent study characterized the in vivo relevance of meprin β for the development of AD in an APPlon-based AD mouse model [30]. Intriguingly, meprin β deficiency recovered the memory deficits of the AD mouse model. Moreover, total Aβ release as well as levels of deposited N-terminal truncated Aβ2-x were significantly decreased in the meprin β knock-out background.

To achieve pathological meprin β expression levels, we aimed to generate meprin β-overexpressing mice to mimic this particular disease condition. We wondered if we could generate an AD mouse model only by overexpressing murine meprin β in the brain and without manipulating endogenous murine APP or its other secretases’ expression. This approach contrasts with many conventional AD research models, as transgenic humanized APP mice, often with manipulations in several other genes, are generally used [45, 46]. Importantly, the use of these transgenic APP models in previous studies led to somewhat biased results, as particularly the insertion of the APPswe mutations at the β-secretase site resulted in the almost exclusive generation of Aβ1-x, whereas Aβ2-x and other N-terminal truncated Aβ peptides are absent [21], thus not properly reflecting the situation in human AD.

Of note, meprin β expression in AD patients’ brains was preferentially detected in astrocytes [29], which is also the cell type that generates the main proportion of N-terminally truncated Aβ2-42 [32]. Therefore, we generated a mouse strain, overexpressing meprin β GFAP-promoter-dependent in astrocytes. Analyzing these meprin β-overexpressing mice, we observed increased amyloidogenic cleavage of the endogenous APP in mouse brain lysates and OBSCs. In addition, we generated a neo-epitope-specific antibody against sAPPβ + Asp, specifically detecting amyloidogenic APP processing by meprin β. Of note, meprin β cleaves APP preferentially between D672 and A673, one amino acid further to the C-terminus compared to the most prominent β-secretase BACE1 (cleaving between M671 and D672). The new antibody specifically detects human and mouse sAPPβ + Asp, but no conventional sAPPβ generated by BACE1.

Although we detected elevated Aβ production in meprin β-overexpressing mouse brains and OBSCs, Aβ deposition in amyloid plaques was not visible. This finding is not surprising, as amyloid plaques have never been observed in mouse brains expressing only endogenous APP. Moreover, the existence of plaques is not necessarily associated with memory deficits in AD. Two independent studies revealed that mutating E693 of APP695 in Tg2576 mice leads to a loss of plaque deposition [47, 48]. Instead, soluble Aβ oligomers were observed to cause cognitive impairments in these mice. Therefore, we conducted behavioral tests with the meprin β-overexpressing mice to characterize explorative behavior, anxiety, and memory function. In doing so, it was observed that meprin β-overexpressing mice exhibit hyperactivity. Of note, also CRISPR/Cas-generated meprin β knock-out mice showed increased hyperactivity [49]. Together with the data obtained in this study, it can be concluded that dysregulation of meprin β leads to hyperactivity. In addition, the meprin β-overexpressing mice showed altered explorative behavior in an open field arena and impaired object location memory, as these mice explored for similar time and with similar frequencies stationary and displaced objects. Interestingly, both impaired object location memory and increased locomotor activity have already been observed in APP transgenic AD mouse models [50, 51]. Thus, the behavior changes might be either the consequence of soluble Aβ accumulation or processing of so far unknown meprin β substrates. To investigate further, N-terminomics will be conducted in a future project to unveil novel substrates of astrocytic meprin β. However, the meprin β overexpression did not lead to altered anxiety, spontaneous alternation or reference memory. Hence, our newly generated mouse model overexpressing meprin β in astrocytes cannot be considered as a classical AD mouse model due to the lack of amyloid plaque deposition and strong memory deficits, but it does show increased AD-like pathological APP processing and AD-associated behavior. In a previous study, the β-secretase BACE1 was also overexpressed in a mouse model with endogenous APP background [52]. The authors observed elevated sAPPβ levels from endogenous APP processing upon BACE1 overexpression in neurons, like we observed sAPPβ + Asp upon meprin β overexpression in astrocytes. However, in that study, Aβ generation was only analyzed when BACE1-overexpressing mice were crossed with transgenic APP mice. Surprisingly, the Aβ formation negatively correlated with the quantity of overexpressed BACE1.

In summary, we generated a mouse model for AD pathology-associated APP cleavage through meprin β overexpression in astrocytes without genetically manipulating the endogenous murine APP sequence and its expression. These meprin β-overexpressing mice showed altered explorative behavior, increased locomotion, and impaired object location memory, which might be caused by elevated Aβ2-x levels. We established methods and tools such as a cultivating system for organotypic brain slices producing N-terminally truncated Aβ from endogenous APP as well as a neo-epitope-specific antibody to detect sAPPβ + Asp, which will likely be helpful for future AD-related studies to examine APP cleavage by different β-secretases and the contribution of different Aβ peptides to AD pathophysiology. Focusing on N-terminally truncated Aβ species in AD research is certainly important, as common mouse models usually express APPswe mutations, from which Aβ2-x is not generated [21], thus contrasting the processes occurring in human AD brains. Hence, Aβ2-x peptides, that show a strong potential to aggregate [28] and are elevated in AD patient brains [20], have likely been underrated so far. In this study, we could show that truncated Aβ species can be generated from endogenous murine APP upon upregulation of the alternative β-secretase meprin β. These N-terminally truncated Aβ species should be considered in AD research.

## Conclusion

In this study, we developed a mouse model for AD-associated APP processing and generation of Aβ2-x through astrocytic overexpression of the alternative β-secretase meprin β. In contrast to conventional APP transgenic AD mouse models generating mainly Aβ1-x [21, 24], our model shows Aβ2-x generation from endogenous APP. These Aβ2-x species are elevated in AD patients’ brains [20], but have been scarcely characterized thus far. Hence, the mouse model offers the opportunity to further study the pathobiochemical properties of Aβ2-x peptides in vivo. Mice and tools generated herein do enable further investigations into a previously largely unnoticed cleavage event and an underrated Aβ form occurring in the brains of AD patients.

### Supplementary Information

Below is the link to the electronic supplementary material.

Supplementary file1 (DOCX 1312 KB)

## Data Availability

The datasets used and/or analyzed during the current study are available from the corresponding author on reasonable request.

## Abbreviations

Aβ: Amyloid-β
AD: Alzheimer’s disease
APP: Amyloid precursor protein
APPlon: London APP
APPswe: Swedish APP
BACE1: β-Site of APP cleaving enzyme 1
CRISPR/Cas: Clustered regularly interspaced short palindromic repeats/CRISPR-associated nucleases
DMEM: Dulbecco’s modified Eagle medium
DMSO: Dimethyl sulfoxide
EDTA: Ethylenediaminetetraacetic acid
EGTA: Egtazic acid
ELISA: Enzyme-linked immunosorbent assay
FDA: U. S. Food and Drug Administration
GPCR: G protein-coupled receptor
HBSS: Hanks’ balanced salt solution
IF: Immunofluorescence
IHC: Immunohistochemistry
IVC: Individually ventilated cage
MTT: 3-(4 5-Dimethylthiazol-2-yl)-25-diphenyltetrazolium bromide
OBSC: Organotypic brain slice culture
PBS: Phosphate-buffered saline
PEI: Polyethylenimine
PFA: Paraformaldehyde
RFU: Relative fluorescent unit
RIPA: Radioimmunoprecipitationbuffer assay
sAPPα: Soluble APP fragment α
sAPPβ: Soluble APP fragment β
sAPPβ + Asp: C-terminally prolonged sAPPβ
SDS: Sodium dodecyl sulfate
SDS-PAGE: SDS-polyacrylamide gel electrophoresis
TBS: Tris-buffered saline
