# Supplementary material for: A novel mouse model for N-terminal truncated Aβ2-x generation through meprin β overexpression in astrocytes
Source: Cell Mol Life Sci. 2024 Mar 13;81(1):139. doi: 10.1007/s00018-024-05139-w (PMC10937767; doi:10.1007/s00018-024-05139-w)
Supplement: Supplementary file 1 — Supplementary file1 (DOCX 1312 KB) [file 18_2024_5139_MOESM1_ESM.docx]

**
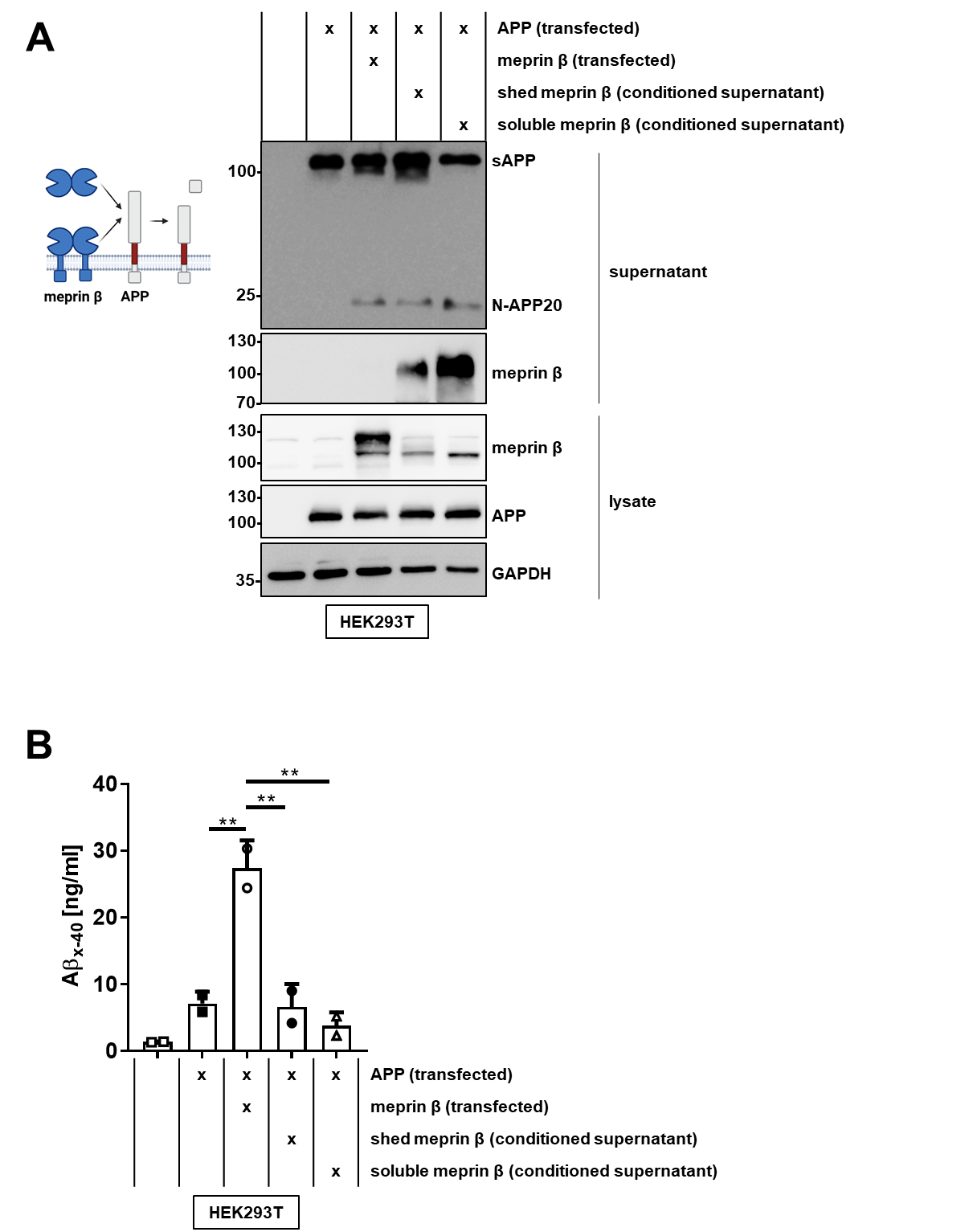
**

**Supplementary Figure S1: Only membrane-bound meprin β exhibits β-secretase activity. (A)** HEK cells were transfected with human APP alone or in combination with human meprin β. After 24 h the medium was changed to serum-free DMEM for 4 h, or it was exchanged with conditioned cell supernatant with shed or soluble meprin β. In order to generate conditioned medium with shed meprin β, HEK cells were transfected with murine ADAM10 and human meprin β. For soluble meprin β, a variant with a stop codon proximal to the transmembrane domain was transfected. Cell lysates and supernatants were analyzed by SDS-PAGE and western blot. **(B)** The culture supernatants of (A) were used for Aβx-40 ELISA. Statistical significance was determined by One-way ANOVA (ns: p > 0.05; *: p ≤ 0.05; **: p ≤ 0.01).


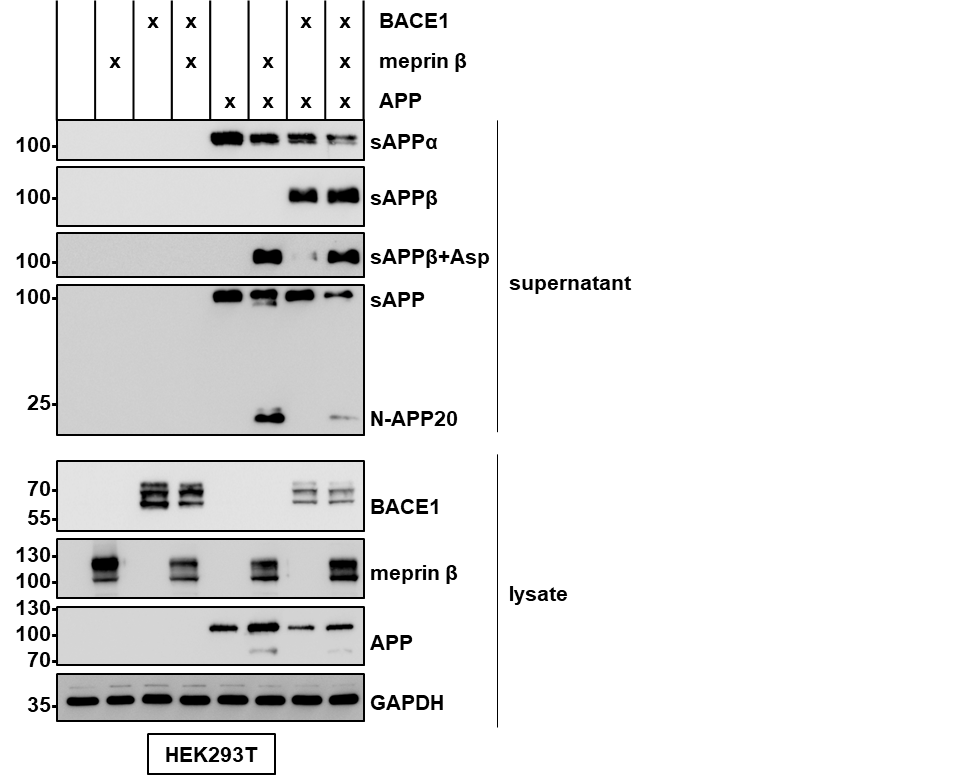


**Supplementary Figure S2: The neo-epitope specific sAPPβ+Asp antibody specifically detects sAPPβ+Asp generated by meprin β.** HEK cells were transfected with human APP (APP) together with BACE1 and/or meprin β. Cell lysates and supernatants were analyzed by SDS-PAGE and western blot.


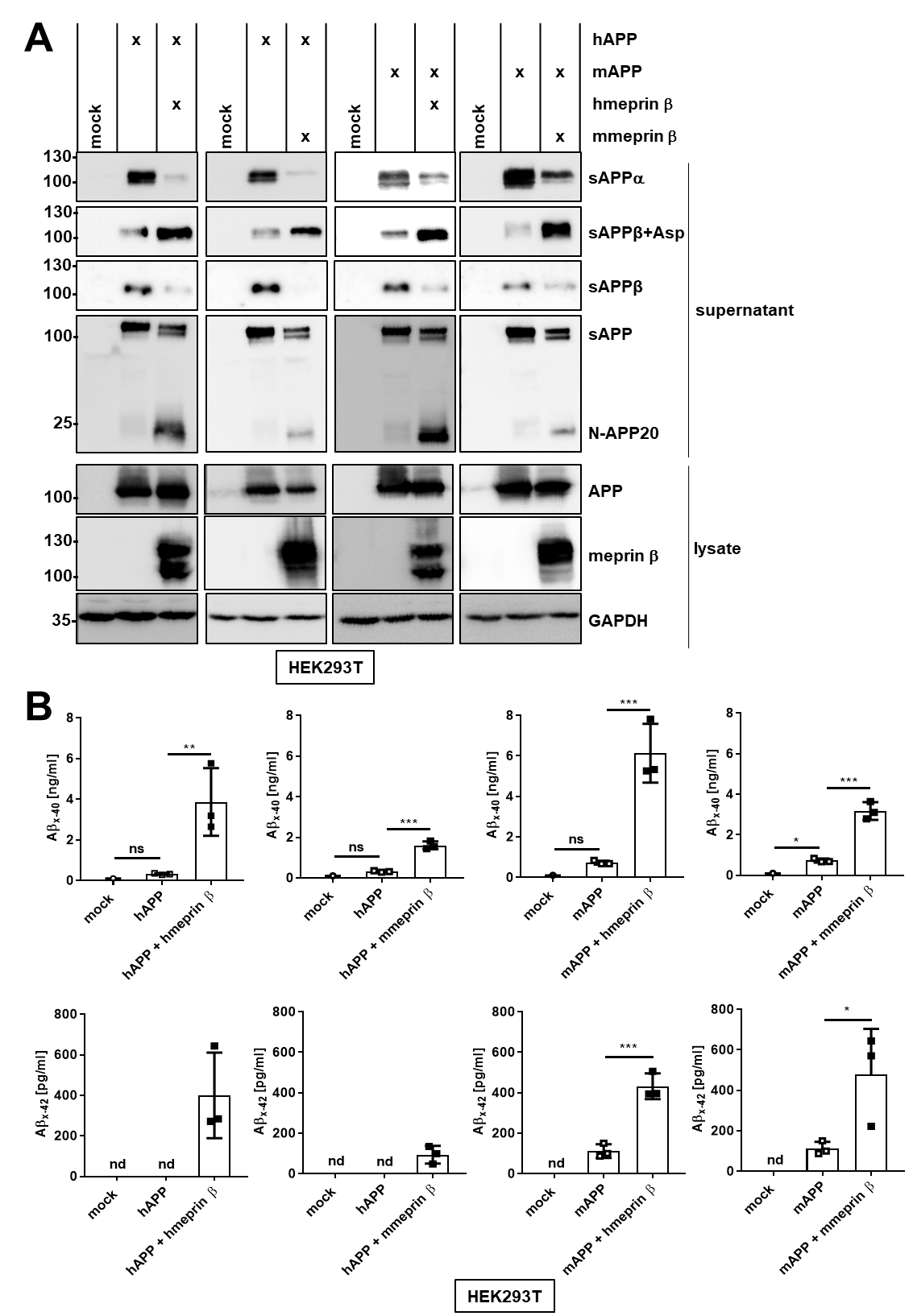


**Supplementary Figure S3: Human and mouse meprin β exhibit β-secretase activity. (A)** HEK cells were transfected with each combination of human APP (hAPP) or murine APP (mAPP) and human meprin β (hmeprin β) or murine meprin β (mmeprin β). Cell lysates and supernatants were analyzed by SDS-PAGE and western blot. **(B)** The culture supernatants of (A) were analyzed using Aβx-40 and Aβx-42 ELISA. Statistical significance was determined by One-way ANOVA (ns: p > 0.05; *: p ≤ 0.05; **: p ≤ 0.01; ***: p ≤ 0.001). nd = not detectable/below the detection limit of the ELISA


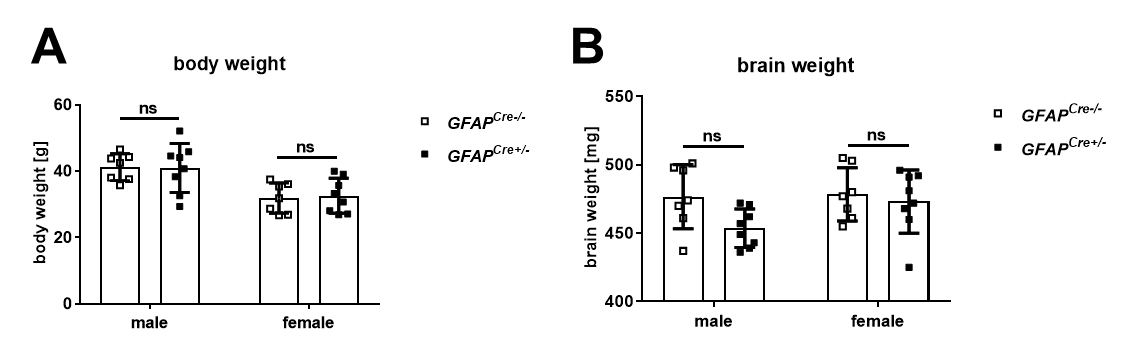


**Supplementary Figure S4: Body and brain weight of mice are not altered upon astrocytic meprin β overexpression. (A)** Body weight of male and female GFAP^Cre^;Rosa26*^Mep1b^*^-HA^ (GFAP^Cre+/-^) and control (GFAP^Cre-/-^) mice (ns: p > 0.05). **(B)** Brain weight of both genotypes. Statistical significance was determined by *t*-test (ns: p > 0.05).
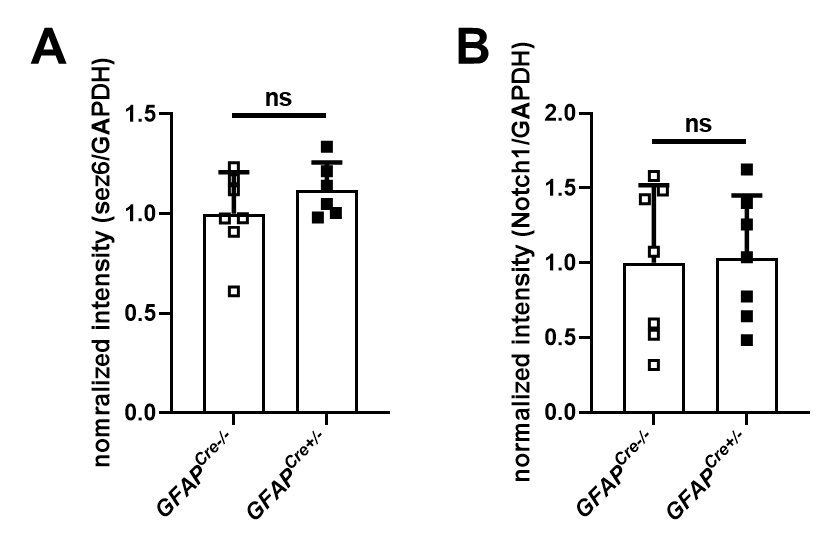


**Supplementary Figure S5: Quantification of sez6 and Notch1 western blot signals.** The sez6 **(A)** and Notch1 **(B)** signals of Figure 3A were quantified and normalized to the GAPDH signal (n=7). The significance level was determined by *t*-tests (ns: p > 0.05).

**
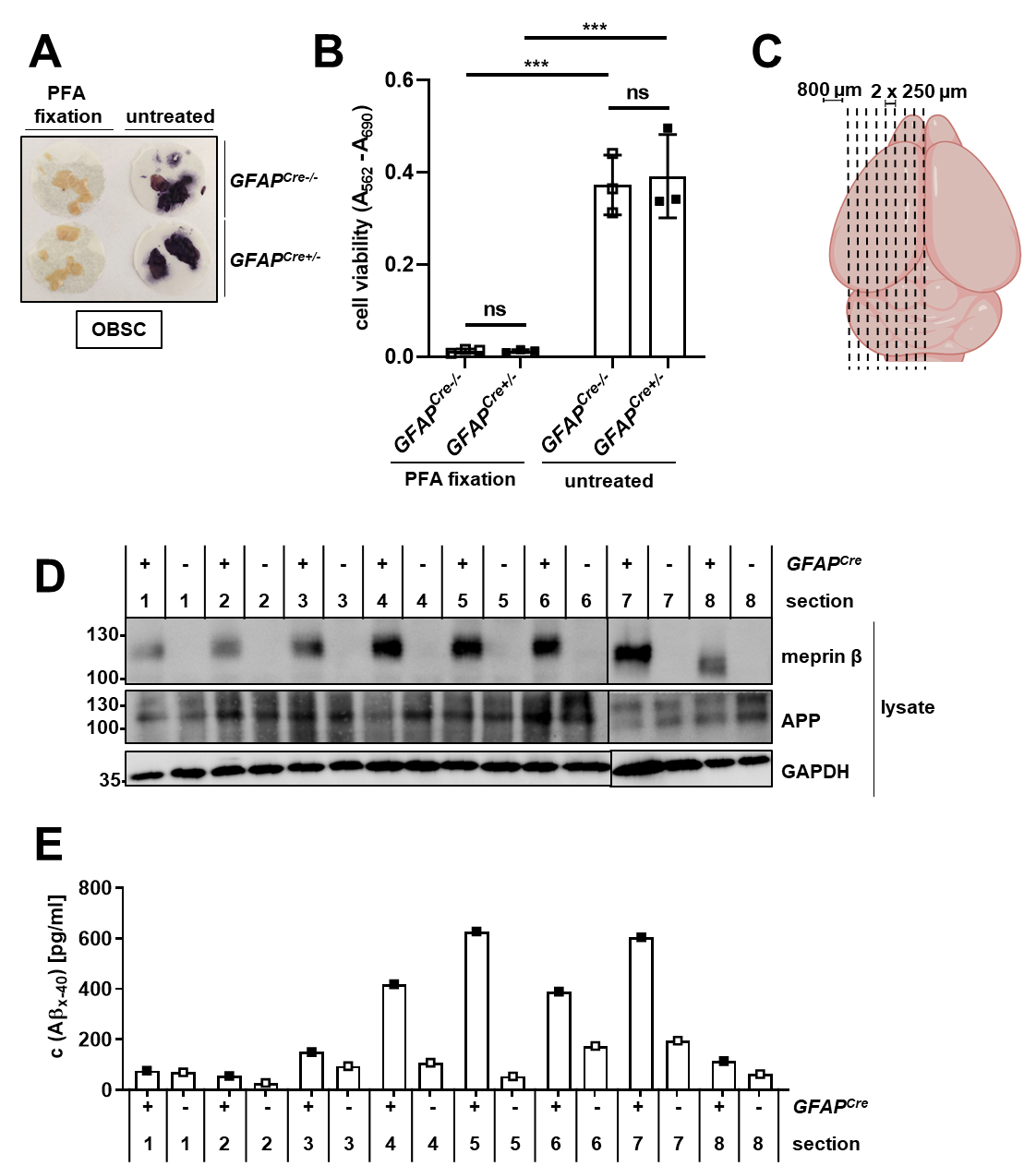
Supplementary Figure S6: OBSCs are viable and produce Aβ upon meprin β expression. (A)** After 20 days in culture organotypic brain slices from GFAP^Cre^;Rosa26*^Mep1b^*^HA^ mice were incubated with serum-free brain slice culture medium containing 0.5 mg MTT/ml. Control slices were fixed with 4% PFA prior to the incubation with MTT. **(B)** The OBSCs treated with MTT were lysed and the viability was quantified by measuring the absorption at 690 nm subtracted from the absorbance at 562 nm. **(C)** The cutting procedure for (D) and (E) is depicted. From the lateral side of one hemisphere of one GFAP^Cre+/-^;Rosa26*^Mep1b^*^-HA^ and one GFAP^Cre-/-^;Rosa26*^Mep1b^*^-HA^ mouse 800 µm were discarded. Then, sixteen 250 µm thick slices were cut to obtain OBSCs of the entire hemisphere. Each two adjacent slices were co-cultivated and referred to as sections 1-8. **(D)** OBSCs were cut as described in (C). The OBSCs were cultivated for 20 d. Lysates and supernatants were analyzed with SDS-PAGE and Western blot. **(E)** The supernatants of (D) were analyzed using the Aβx-40 and Aβx-42 ELISA.

**
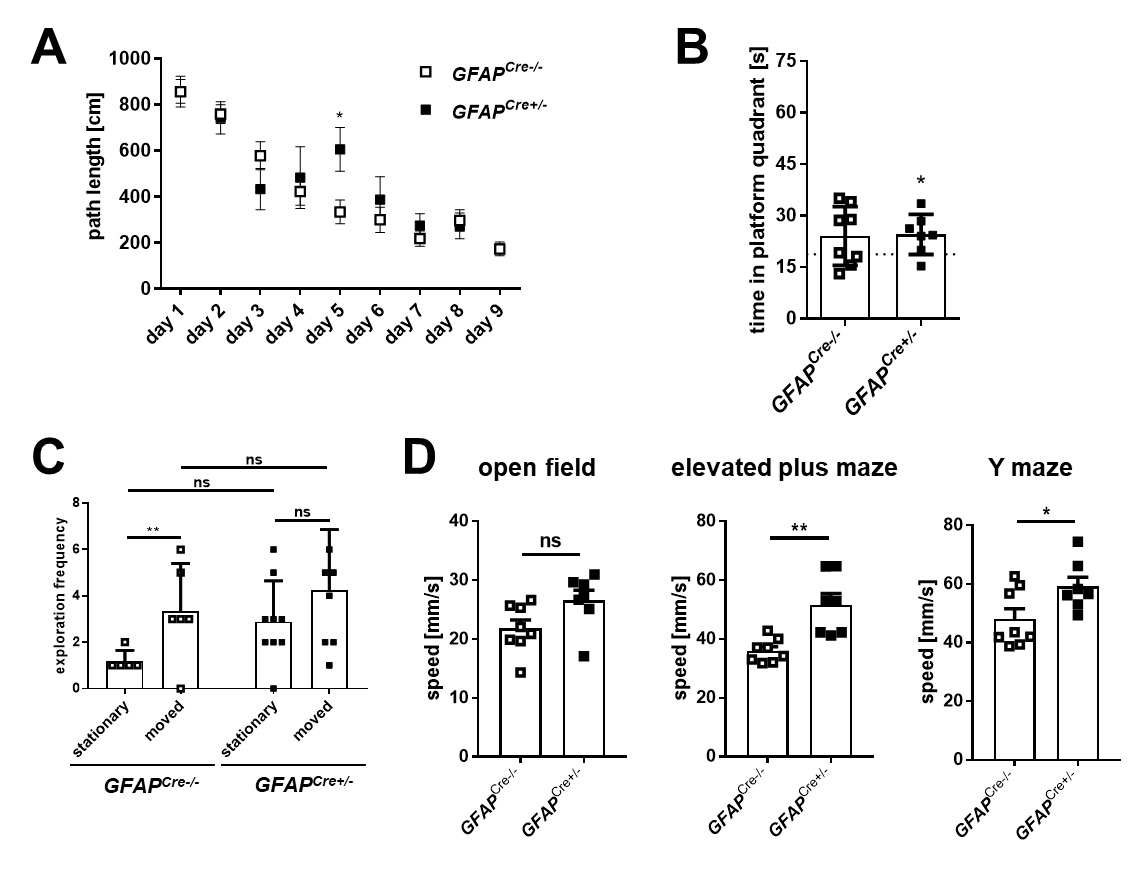
**

**Supplementary Figure S7: Additional data of the mouse behavior tests. (A)** Open field water maze. The average path length is plotted (4 trials each day, 8 and 7 mice). A Two-way ANOVA was used to determine the significance level (ns: p > 0.05; *: p ≤ 0.05). Path length shortening, while learning, shows a similar trend comparing control and GFAP^Cre+/-^ mice. **(B)** Open field water maze. On day nine, the platform was removed before the probe trial and time spent in the correct quadrant was recorded. Here data of both groups are plotted against the chance time to be spent in each of the quadrants (total trial time/number of quadrant = 18.75 s). Both groups show average time above chance, t-test gave significance only for GFAP^Cre+/-^ **(C)** Spatial object recognition. The graph shows the exploration frequency, acquired tracking the nose in the object area, during a trial with one object located in the familiar position and one displaced to a new location. GFAP^Cre-/-^ mice visited a higher number of times the object in the new location while GFAP^Cre+/-^ explored stationary and moved objects with a similar frequency. **(D)** Open field test, elevated plus maze and Y maze. The average speed acquired (mm/s) was always higher for GFAP^Cre+/-^ comparing with controls. The significance level was determined by t-test (ns: p > 0.05).
